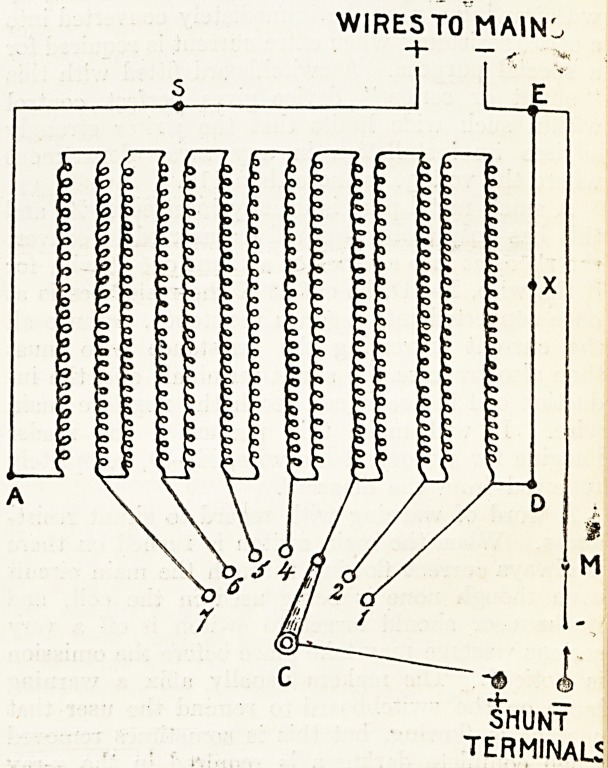# The X-Rays

**Published:** 1912-09-28

**Authors:** Alfred C. Norman

**Affiliations:** House Surgeon at Durham County Eye Infirmary.


### ELECTRICITY IN MODERN MEDICINE.*

### XVIII.—The X-Rays (*Continued from page 564*).

By ALFRED C. NORMAN, M.D. Edin., House Surgeon at Durham County Eye Infirmary.

There is no need to describe in detail the various accessories on the switchboard. The makers will see that they are selected and arranged so as to get the best possible results from the coil and interrupter. But it may assist the beginner to understand his apparatus if we consider here a few of the practical points in connection with these accessories.

*The Resistances.*—We have already seen that the wire of which a resistance coil is made must be sufficiently thick to carry all the current required of it without becoming dangerously hot, and that it must be long enough to afford all the resistance ever likely to be needed in its own branch of work. It has been shown why a cautery resistance must contain a few turns of thick wire, why a resistance for galvanic treatment should consist of many thousands of turns of wire as thin as a hair, and why a resistance for lighting medical lamps must be between the two. It now remains to consider the large resistance used in controlling the current through the primary winding of the induction coil.

For currents above 110 volts it is advisable to use a shunt resistance. By this means we can vary the voltage as well as the amperage passing through the induction coil; whereas, in the case of a series resistance, the voltage must always be that of the supply, though the amperage can be varied by increasing or diminishing the resistance in circuit.

One advantage of a shunt resistance is that we need not use a higher voltage than will just give us the required amperage through the primary coil. Using a modern coil with a high self-induction and a hard tube, the writer has never been able to satisfy himself that there is a greater tendency to reverse current when the voltage in the primary is high; but with a soft tube or with a coil of low self-induction this tendency undoubtedly becomes manifest, and it is then of great advantage to be able to keep the voltage low by means of a shunt resistance. Tubes certainly last longer when used with a shunt resistance, and this is probably due to the fact that we have more control over them during the stages when they are new and soft. Another advantage of this type of resistance is that there is less sparking in the interrupter owing to the latter, with the coil, being in a shunt circuit.

A disadvantage of the shunt resistance is that the current passing through the main circuit does not all pass through the coil, so that we have to pay for a certain amount of current that we do not actually use. But electricity is cheap in these days, and its cost is a matter of small importance compared with the above-mentioned advantages.

When the supply is a current of less than 110 volts it is not so likely to produce reverse current, and it can be easily controlled by a series resistance; hence the latter should be installed under these circumstances. If we use a series resistance there is, of course, no need for a volt-meter on the switchboard, for we know that the voltage passing through the coil must be the same as that of the main supply.

Fig. 1 illustrates diagrammatically the connections of a shunt rheostat arranged for use on a supply of 220 volts. The wire must be thick because it will be required to carry from 7 to 10 amperes, and for our purpose it must have a total resistance of about 30 ohms. But to obtain 30 ohms resistance with such thick wire we must use many yards of it, and this is generally arranged in coils at the back of the switchboard—making it a very cumbersome piece of apparatus.

The principle of this resistance is exactly similar to that shown at Fig. 2, page 510 of The Hospital for February 17, except that in the present instance current is collected by a crank, C, which can be turned so as to make contact with a series of studs, 1—7, instead of by a sliding contact. These studs are connected at various points with the coils of resistance wire, and are so arranged that by turning the crank we are able to obtain voltages varying from about 170 when the crank is in contact with stud No. 7, to 0 when it is on stud No. 1.

Since the total resistance of the wire (Fig. 1) is 30 ohms, there will be a current of about $7\frac{1}{2}$ amperes

* Previous articles appeared on Nov. 11, 25, Dec. 9, 30, Jan. 13, 27, Feb. 17, March 9, 30, April 20, May 4, 25, June 8, July 6, and Aug. 3, 17, 31.

traversing the main circuit whenever the main switch is on. This current enters at the main wire marked + (at the top of the diagram) and leaves at the main wire marked – after having traversed the coils of resistance wire, A—D. The voltage between the two shunt terminals (at the bottom of the diagram) will be determined by which of the studs, 1—7, the crank, C, is in contact with, and when the induction coil is connected with the shunt terminals the amperage flowing through it can be perfectly controlled by varying the voltage.

It occasionally happens that a tube becomes so hard that we are unable to get sufficient current through it from a shunt resistance constructed for working with ordinary tubes, and this would undoubtedly be a great disadvantage; but it is now the custom to arrange the resistance so that it can, by withdrawing a plug, be immediately converted into a series resistance when extra current is required for a special purpose. A switchboard fitted with this "shunt or series" device gives perfect control within such wide limits that the writer strongly advises its installation in any *x*-ray department where the voltage is more than 110.

A small metal plug is usually inserted at X, and this has only to be removed or inserted to convert the rheostat into a series or a shunt one at will, for if the wire, E—D, be cut at X the resistance is at once converted into a series resistance, because all the current traversing the resistance wire must then also traverse the shunt terminals and the induction coil before it can reach the negative main wire. It will make this plainer if the reader imagine for a moment the wire, E—D, completely removed from the diagram.

A word of warning with regard to shunt resistances. When the main switch is turned on there is always current flowing through the main circuit even though none is being used in the coil, and if the user should forget to switch it off a very serious wastage may take place before the omission is noticed. The makers usually affix a warning lamp on the switchboard to remind the user that current is flowing, but this is sometimes removed when complete darkness is required in the *x*-ray room, and then it is an easy matter to forget to turn off the main switch. For as soon as current has been shut off from the coil by turning the crank, C, to stud No. 1, there is nothing to indicate that current is still flowing in the main circuit.

The *small sliding rheostat* for controlling the speed of the motor has to carry less than 1 ampere of current, so it is constructed of fine wire—a comparatively small quantity of which furnishes the required resistance of about 100 ohms.

The *ampere-meter* and *volt-meter* should be of the dead-beat type—that is to say, the indicating needle should come to rest almost as soon as the current is switched on, instead of oscillating to and fro for several seconds, as usually happens in the cheaper kinds of meter.

It may be noted here that an ampere-meter has very little resistance of its own, consequently it must always be connected in series with some apparatus with sufficient resistance to protect it. For instance, to connect an ampere-meter across the two wires of the main supply would be tantamount to short-circuiting the current through the meter—with disastrous results to the meter and to the fuses of the supply. On the other hand, a volt-meter has a high resistance, for it is intended to be connected directly between the positive and negative wires of the supply for the purpose of registering the voltage (difference of potential) between them. The volt-meter really forms a small shunt circuit on its own account through which only a small fraction of an ampere is allowed to pass by reason of the high resistance of the meter.

In the diagram (Fig. 1) the ampere-meter could be conveniently fixed at M, the wire being divided at that point and the terminals of the meter connected with the cut ends. The volt-meter would be connected directly with the shunt terminals by two separate wires—not shown in the diagram.

The *main-switch* should be fitted with a spring knife-blade arrangement for producing a quick break, otherwise there is a risk of producing a powerful "arc" when the current is broken. This is due to self-induction in the primary circuit, which greatly increases the voltage at the moment the current is switched off and forces the latter to jump or "arc" across a considerable air-gap between the contacts of the switch.

We have seen that when a *continuous* (*i.e.* unidirectional) current from the street mains is available for *x*-ray work it is a simple matter to send such a current directly through the switchboard, interrupter, and induction coil, and so transform it into a current of exceedingly high voltage, perfectly suitable for exciting an *x*-ray tube.

When the available supply is an *alternating* current, however, the problem is more difficult, for this current must be made unidirectional before it is sent through the induction coil, otherwise a very high proportion of reverse current will be generated. There are many so-called current rectifiers on the market designed to make an alternating current unidirectional by suppressing one of its phases, but none of them are really satisfactory, and it is far cheaper in the long run to install a motor generator outfit, as described on page 509 of The Hospital for February 17. The dynamo of this little outfit is rotated by an alternating current motor and furnishes a continuous current of 110 volts, which is an ideal current for sending through the induction coil.

In districts where there is no public supply of electricity a generator outfit driven by a small oil engine can be installed. These little outfits are now quite reliable and give very little trouble. The dynamo is exactly similar to the one mentioned above, but it is driven by an oil engine instead of by an alternating current motor.

Portable accumulators are sometimes used in places where there is no public supply of current, but they should only be adopted as a last resort. It is a troublesome business sending them away to be re-charged. and they do not furnish sufficient power for modern *x*-ray work.

(*To be continued.*)

## Figures and Tables

**Figure f1:**